# Broken Heart Syndrome: Evolving Molecular Mechanisms and Principles of Management

**DOI:** 10.3390/jcm12010125

**Published:** 2022-12-24

**Authors:** Yashendra Sethi, Hamsa Murli, Oroshay Kaiwan, Vidhi Vora, Pratik Agarwal, Hitesh Chopra, Inderbir Padda, Manasa Kanithi, Mihaela Simona Popoviciu, Simona Cavalu

**Affiliations:** 1 PearResearch, Dehradun 248001, India; 2 Department of Medicine, Government Doon Medical College, Dehradun 248001, India; 3 Department of Medicine, Lokmanya Tilak Municipal Medical College, Mumbai 400022, India; 4 Department of Medicine, Northeast Ohio Medical University, Rootstown, OH 44272, USA; 5 College of Pharmacy, Chitkara University, Rajpura 140401, Punjab, India; 6 Richmond University Medical Center, Staten Island, NY 10310, USA; 7 College of Osteopathic Medicine, Michigan State University, East Lansing, MI 48824, USA; 8 Faculty of Medicine and Pharmacy, University of Oradea, P-ta 1 Decembrie 10, 410087 Oradea, Romania

**Keywords:** Broken Heart Syndrome, Takotsubo cardiomyopathy, Happy Heart Syndrome, stress cardiomyopathy, Apical Ballooning Syndrome, Takotsubo Syndrome

## Abstract

Broken Heart Syndrome, also known as Takotsubo Syndrome (TS), is sudden and transient dysfunction of the left and/or right ventricle which often mimics Acute Coronary Syndrome (ACS). Japan was the first country to describe this syndrome in the 1990s, and since then it has received a lot of attention from researchers all around the world. Although TS was once thought to be a harmless condition, recent evidence suggests that it may be linked to serious complications and mortality on par with Acute Coronary Syndrome (ACS). The understanding of TS has evolved over the past few years. However, its exact etiology is still poorly understood. It can be classified into two main types: Primary and Secondary TS. Primary TS occurs when the symptoms of myocardial damage, which is typically preceded by emotional stress, are the reason for hospitalization. Secondary TS is seen in patients hospitalized for some other medical, surgical, obstetric, anesthetic, or psychiatric conditions, and the dysfunction develops as a secondary complication due to the activation of the sympathetic nervous system and the release of catecholamines. The etiopathogenesis is now proposed to include adrenergic hormones/stress, decreased estrogen levels, altered microcirculation, endothelial dysfunction, altered inflammatory response via cardiac macrophages, and disturbances in the brain-heart axis. The role of genetics in disease progression is becoming the focus of several upcoming studies. This review focuses on potential pathophysiological mechanisms for reversible myocardial dysfunction observed in TS, and comprehensively describes its epidemiology, clinical presentation, novel diagnostic biomarkers, and evolving principles of management. We advocate for more research into molecular mechanisms and promote the application of current evidence for precise individualized treatment.

## 1. Introduction

Takotsubo cardiomyopathy, (also known as Tako-tsubo Syndrome, Apical Ballooning Syndrome, stress cardiomyopathy and Broken Heart Syndrome) is characterized by an acute akinesia/hypokinesia of the mid and apical segments of the left ventricle and hyperkinesia of the basal segment, leading to left ventricular dysfunction [1]. The name of the condition ‘Takotsubo’ derived from the characteristic ‘ballooning’ similar to a Japanese octopus trap. In 1990 and 1991, Sato and Dote coined the term Takotsubo (tako = octopus, tsubo = a pot) to describe the left ventricular silhouette during systole in five patients with clinical features of myocardial infarction but no obstructive coronary artery disease. More recently, atypical forms of this condition involving hypokinesia of the basal segment and various other types of wall motion abnormalities have been described [2,3].

Around 2% of individuals presenting with clinical manifestations of ACS have been reported to have TS (up to 10% if only women are considered) [4]. Unfamiliarity with the disease is a major contributor to its underdiagnosis and underestimation of its prevalence. More people are learning about TS and are accessing early invasive coronary angiography, so the condition is being diagnosed at earlier stages. The incidence of TS rose dramatically between 2006 and 2012, by a factor of nearly 20, as reported by Minhas et al. [5]. Similarly, a study by Murugiah et al. demonstrated rising TS hospitalization rates [6]. The study found that the rate of Primary TS hospitalizations rose from 2.3 in 2007 to 7.1 in 2012 for every 100,000 people. When measured in terms of hospitalizations per 100,000 person-years, the incidence of ‘Secondary TS’ rose from 3.4 in 2007 to 10.3 in 2012. According to global studies, 85% to 90% of patients with TS were women between the ages of 65 and 70 [7,8]. Templin et al. recently reported that out of 1750 patients with TS, 89.8% were female with a mean age of 67 [9]. The cumulative incidence of recurrence was 1.2% at six months and 5% at six years, as reported by Singh et al. [10]. The reported annual recurrence rate is 1.5%.

**Figure 1:**
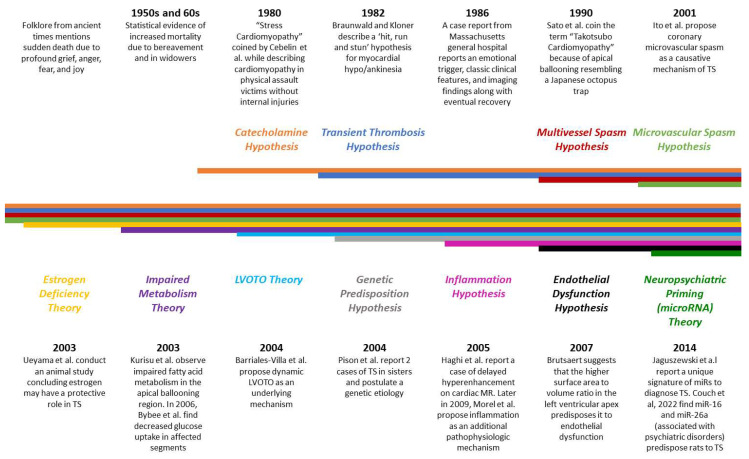
Timeline—showing the landmark research developments over the years for Broken Heart Syndrome [12,13,16,19,20,21,22,23,24,25,26,27,28,29,30,31].

The exact pathophysiology underlying TS remains an enigma (Figure 1). In the 2000s, postulated theories included catecholamine-induced multiple epicardial spasms, microvascular spasms, and direct myocardial injury [11]. However, the role of catecholamines was recognized by Cebelin et al. well before the term “Takotsubo cardiomyopathy” was coined [12]. Another prominent mechanism proposed was a dynamic left ventricular outflow tract obstruction, which was later thought to be a compensatory mechanism rather than underlying pathophysiology [11,13].

Endothelial dysfunction due to falling estrogen levels and mental stress due to the trigger was a hypothesis used to explain the higher prevalence in postmenopausal women, further supported by an animal study [14,15,16]. A transient, self-aborting myocardial infarction with rapid thrombus dissolution was another prominent theory [17]. The possibility of a genetic predisposition was also starting to be explored [18]. The last decade has seen explosive progress in our understanding of the disease, which has opened doors to new targeted treatment modalities. A combination of various pathophysiological mechanisms is considered more accurate than any one mechanism alone.

There has been a significant lead in evidence for Broken Heart Syndrome over the past few years and thus the current review attempts to compile and appraise the evolving molecular mechanisms and principles of management for TS.

## 2. Etiopathogenesis of TS

The pathophysiology of Takotsubo Syndrome is elusive, the specificities of which are still being investigated, which makes the diagnosis and treatment more strenuous. Several mechanisms for TS development have been proposed. A plethora of research suggests that the primary cause of TS is a physically or emotionally stressful trigger [11]. The secondary cause includes medical, surgical, or psychiatric emergencies. Several diverse circumstances can predispose, trigger, and eventually result in TS [32]. These circumstances can be classified into three types, i.e., predisposing factors, triggers/stressors, and pathogenic mechanisms [33].

### 2.1. Predisposing Factors 

Some of the modifiable risk factors for Takotsubo Syndrome were researched in a systematic review. In the absence of any coronary artery disease, the study found a high prevalence of cardiovascular risk factors in patients with TS. These risk factors included obesity (17%), smoking (22%), and pre-existing medical conditions such as hypertension (54%), dyslipidemia (32%), and diabetes (17%). Other comorbidities included psychological disorders (24%), pulmonary diseases (15%), malignancies (10%), neurologic diseases (7%), chronic kidney disease (7%), and thyroid diseases (6%) [34]. These factors were similar in frequency, as seen in patients with acute MI. These findings were consistent with Summer et al.’s prior observations, which demonstrated that the majority of patients with TS also had at least two of the aforementioned predisposing risk factors [35]. According to Martin et al., these cardiovascular risk factors can lead to endothelial dysfunction [36] which then can be another predisposing component [37].

The current evidence suggests gender be a non-modifiable risk factor for TS. Several reviews show a marked gender discrepancy with a higher prevalence of TS in females, especially postmenopausal women. This association can be explained by the effect of sex hormones on hypothalamo-sympathoadrenal outflow [38] and coronary vasoreactivity [39] and supports the hypothesis that estrogen deficiency plays a major role in the pathophysiology of TS.

### 2.2. Role of Stressors

Despite the ambiguity in the pathogenesis of TS, the current evidence agrees upon the impact of stressors in causing TS. Sudden somatic and/or emotional stress causes a rise in the levels of catecholamine, resulting in transient left ventricular dysfunction [11]. Stressors can be further classified into two groups: physical and emotional stresses; although the various triggering events recorded to date are diverse. A study showed that in patients with TS, about 39% experienced a triggering emotional stressor whereas 35% experienced a physical stressor [34]. Somatic or physical stressors researched to precede TS are vigorous exercise, hyperthyroidism, alcohol/opiate withdrawal, and postoperative pain, etc. [40]. Recent research has also discovered the intimate connection between TS and medical conditions such as ischemic stroke, subarachnoid hemorrhage (SAH), and epileptic seizures [10,41,42,43]. A study correlated cerebrovascular accidents with ten times increased likelihood of developing TS [11]. Thus, supporting a pathogenesis involving increased catecholamines as proposed by Greco et al. in the 1980s [44]. Emotional stressors can vary vastly and include grief (death or accident of a loved one), receiving unexpected or bad news (being diagnosed with a serious illness), fear (armed robbery, public speaking), change in residence, anger (fight with a partner), relationship discord (break-up or divorce), financial troubles (layoff, demotion), and being bullied. The importance of acute emotional stress in triggering TS led to the origination of name Stress-Induced Cardiomyopathy and Broken Heart Syndrome [34,40].

### 2.3. Pathogenic Mechanisms 

The etiopathogenesis can be explained by cardiovascular and neuropsychiatric mechanisms.

#### 2.3.1. Cardiovascular Mechanisms

The major cardiovascular hypotheses explaining the pathophysiology of TS have been categorized into vascular, myocardial, or both. In the vascular category, the cause of TS can be attributed to acute coronary spasm of multiple vasculatures, aborted myocardial infarction with spontaneous recanalization, or acute increased ventricular afterload. Multivessel coronary vasospasm was seen in 5–10% of individuals spontaneously and in 28% of individuals when provoked [45]. However, the argument against this hypothesis presents the role of dobutamine (vasodilator with minimal vasospastic effect) and epinephrine (coronary vasodilatory effect) in inducing TS. Another proposed vascular hypothesis is increased afterload causing TS. Increased afterload leading to TS can occur due to various reasons, but is mainly due to hypertension [31,45].

Under the myocardial category, the cardiac cause or manifestation is acute left ventricular outflow tract obstruction (LVOTO) and direct catecholamine-mediated myocardial stunning. Several studies support the LVOTO hypothesis which also leads to symptoms commonly associated with TS. For example, LVOTO compromises the forward stroke volume, and oxygen supply-demand mismatch and can lead to hypotension or cardiogenic shock in severe cases [8,11]. A research study found 25% of individuals with TS manifested LVOTO [11]. Risk factors predisposing patients to TS due to LVOTO were small left ventricle or localized sigmoid septum. Arguments against this hypothesis state that LVOTO cannot explain the apical-sparing and basal patterns seen in several patients with TS. Furthermore, the right ventricle is also involved in several cases which cannot be explained by an LVOTO hypothesis [11]. Therefore, LVOTO can be stated as a complication of TS and not a cause. 

#### 2.3.2. Neuropsychiatry and TS

Takotsubo Syndrome is believed to be a consequence of several psychiatric and neurological conditions as it is closely linked with various organs, especially the brain [40]. Depression and chronic stress have been linked to anatomical alterations in the cerebrum, including decreased hippocampal volume and gray matter loss which have been connected to changes in the HPA axis in response to stress [46]. Women diagnosed with TS were more likely to have a history of chronic anxiety disorder preceding the TS event compared to controls or patients with acute MI [35].

A study by El-Sayed et al. demonstrated that patients with anxiety and mood disorders show increased susceptibility to Takotsubo Syndrome presumably because they are linked to an increased likelihood of stressful occurrences [47]. If this is correct, it adds to the notion that Takotsubo encompasses both mental and somatic medicine, emphasizing the underlying relationship between the two. Two recent reviews also found that chronic stress and depression are associated with significant odds of developing Takotsubo Syndrome [47,48]. A study by Corrigan, Frank E 3rd et al., demonstrated that underlying psychiatric disorders or their exacerbation can increase an individual’s susceptibility to developing TS in response to a strong emotional and/or somatic stressor [49]. 

## 3. Underlying Molecular Mechanisms and Recent Updates

**Figure 2:**
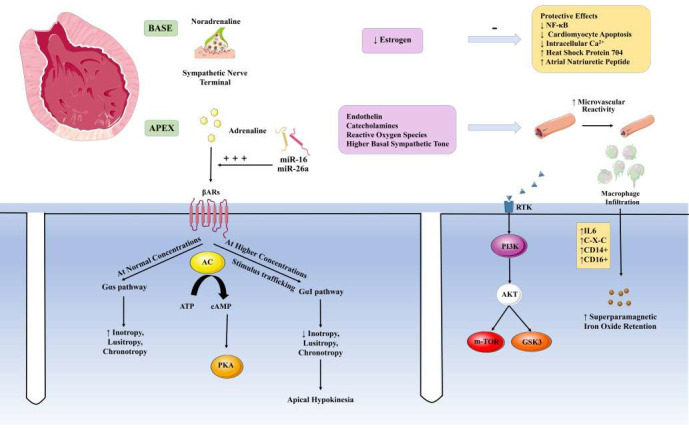
Molecular mechanisms for Broken Heart Syndrome. Parts of the figure were drawn using pictures from Servier Medical Art (smart.servier.com) [accessed on 2 November 2022], provided by Servier, licensed under a Creative Commons Attribution 3.0 unported license. (https://creativecommons.org/licenses/by/3.0/).

The understanding of the molecular and cellular mechanisms behind Broken Heart Syndrome has evolved predominantly over the past few decades. This has enabled better understanding of the disease process (Figure 2) and has aided in planning a more individualized treatment plan as per the underlying mechanism involved.

### 3.1. Role of the Adrenergic System and Myocardial Survival Pathways

The βARs play a pivotal role in regulating the response of cardiac myocyte to catecholamines in times of stress and exercise. The distribution of βARs is not the same throughout the cardiac myocardium. It follows a spatial organization with concentration at the cardiac apex as shown in Figure 2 [31]. The concentration of β1ARs was found to be almost four times more than β2ARs in the human myocardium. Moreover, a downregulation of the β1ARs subpopulation was reported in a failing left ventricle [50]. At normal concentrations of adrenaline, both β1 and β2 receptors function via the Gαs pathway, leading to downstream activation of adenylate cyclase and an increase in cAMP, thereby resulting in an increase in inotropy, chronotropy and lusitropy. However, at higher concentrations of circulating adrenaline, as seen in TS, the receptor signals via the Gαi pathway through a process called ‘Stimulus trafficking’ or ‘Biased agonism’. This causes direct and indirect inhibition of the Gαs activity thereby leading to a decreased inotropy, chronotropy, and lusitropy [31,51]. This is believed to contribute to apical hypokinesia, as seen in TS. This model was confirmed by selective inactivation of the Gi pathway using pretreatment with pertussis toxin thereby preventing the effect of increased levels of adrenaline [52].

These mechanisms of adrenergic action contribute ‘transient’ severe wall motion abnormalities in TTS patients, which suggests the protective role of the mechanisms in maintaining myocardial integrity. Myocardial protection could be triggered by two distinct mechanisms. The first includes protective mechanism is linked with adrenoceptors. When exposed to supra-physiological epinephrine concentrations, beta 2-adrenoceptors switch from Gs to Gi coupling, resulting in a negative inotropic response that mitigates the extent of acute myocardial injury. The second is phosphoinositide 3-kinase/protein kinase B (AKT) survival pathway, which is transiently activated during the acute phase of TTS. Coronary angiogenesis and cardiac growth after birth both rely on the kinase AKT. Mechanistic target of rapamycin and glycogen synthase kinase 3 (GSK3) are two of its downstream targets that are known to play important roles in regulating metabolism, cell proliferation, and even cell survival. To keep cells alive, multiple mechanisms work in tandem, including direct apoptosis inhibition, inhibition of proapoptotic transcriptional factors, augmentation of antiapoptotic transcriptional factors, and inhibition of the GSK3 to improve cell metabolism. Several clinical studies demonstrating ‘inverse perfusion-metabolism mismatch,’ which is commonly observed during myocardial stunning, confirm that myocardial function down-regulation is a protective mechanism caused by a severe reduction in perfusion [53,54,55].

### 3.2. Role of Estrogen

Estrogen plays an important role in TS due to its cardioprotective action (Figure 2). Estrogen allows for a decreased sensitivity of the β1ARs to ischemic injury, inhibition of apoptosis of the cardiac myocytes, inhibition of NF-κB activation, and decreased intracellular calcium levels with increased clearing [53]. Estrogen has also been researched to increase the level of substances such as heat shock protein 704 and Atrial Natriuretic Peptide thereby conferring its cardioprotective action [56]. Research on lactating women suffering from TS has also pointed to a possible cardioprotective action of estrogen which is inhibited due to the effect of elevated prolactin on the gonadal axis [57]. Estrogen has also been found to prevent cardiac hypertrophy by inhibition of calcineurin [58]. In a study by Brenner et al. involving 17 TS and 16 MI patients, a significant elevation of E2 concentration was found in postmenopausal women with TS compared to ones with MI. However, an assessment of sex hormones by Moller et al. on 57 patients each with TS and MI found no significant difference in E1 concentration between both female patients [59].

### 3.3. Genetic Polymorphisms 

The pathophysiologic and molecular mechanisms are largely unknown and the limited studies conducted lack significant samples for a conclusive declaration. An excess of catecholamines is said to be the possible pathophysiologic phenomenon that contributes to the development of TS [60]. This is mediated by the β1 and β2 receptors coupled by a G protein subunit. Hence, polymorphisms in these components have been found to contribute to the genetic component of TS.

The ADR1 and ADR2 genes code for the β1 and β2 receptors, respectively. Polymorphism in these genes contributes to the increased sensitivity of the heart to catecholamines and adrenergic stress. Common β1 polymorphisms, that include Arg389 and Ser49 are proposed to play a role by causing a functional alteration or a gain of function mutation. In a case-control study by Vriz et al., some associations have been found between β1 adrenergic receptor polymorphisms and the occurrence of TS. However, no significant difference was found with β2 receptor variation [61]. A possible polymorphism at 322–325 in the ADRA2C receptor, that codes for Norepinephrine release from the nerve terminals may alter the receptor coupling and signaling, thereby contributing to an impaired release of Norepinephrine and TS pathogenesis [62]. A significant difference in cases and controls was found for GRK5 gene polymorphism with an increased prevalence of the Leucine (L41) over the Glutamine variant. The same research concluded no significant variation in the frequency of β1, β2, and GNAS polymorphisms. However, similar research involving a large Australian cohort of 92 patients showed no significant association of the GRK5 gene polymorphism. Similarly, no significant association of the β1 and β2 adrenergic receptors along with the ER alpha and COMT receptors was found [63].

Gene Polymorphism in the BAG3 gene that contributes to endothelial cell apoptosis regulation may contribute to the pathophysiology of TS by affecting the coronary microcirculation [64]. Another large case-control study with 461 patients and 403 controls demonstrated no significant association with the BAG3, GRK5, or AR3 gene [65]. With a negative association on polymorphisms, studies focusing on Genome-Wide Exon Sequencing have evolved to predict the mechanism of development [66].

### 3.4. MicroRNAs and Brain Heart Axis 

Circulating microRNAs have been indicated in the possible pathogenesis of TS by sensitizing the heart to an adrenergic stimulus. In a study on rat cardiomyocytes, miR-16 and miR-26a were found to enhance the inotropic effect of adrenaline along with ventricular basal hypercontractility and apical hypokinesis [67]. Jaguszewski et al., in their study to find a novel signature to differentiate TS from ST elevation MI, also found a significant elevation of miR-16 and miR-26a as compared to healthy controls along with let-7f as compared to STEMI patients [30]. A study by Avenia et al. points to a novel pathway of BAG3 upregulation as a result of epinephrine. The study on 70 patients with TS found a g2252c polymorphism in the BAG-3 gene that resulted in a loss of microRNA-371a-5p binding, thus leading to a modified response to epinephrine [68]. 

A higher prevalence of neuropsychiatric disorders has been found in patients with TS [9]. The recent literature points to a possible link between anxiety and TS due to increased sympathetic activity [69]. Jaguszewski et al., in an attempt to find a signature of circulating miRNAs for TS detection, found depression and stress-related miR-16 and 26a to be positively correlated [30]. MRI analyses have also found a decreased thickness in the insular region and cingulate cortex along with reduced structural brain connectivity in the left parahippocampal gyrus and amygdala, both hippocampi, right putamen, and the left superior temporal pole [70]. In a retrospective analysis by Heistand et al., higher activity in amygdala was found to be associated with an increased risk of TS [71].

### 3.5. Cardiac Macrophages

A catena of studies has shown monocyte and macrophage infiltration of the myocardium in TS (Figure 2). Although the functional significance of these results is still obscure, recent experimental studies have highlighted some role of cardiac macrophages enhancing our molecular understanding of the disease process. Liao et al., in their study on mice, demonstrated that a single dose of isoproterenol (ISO) caused a cardiomyopathy-like TS phenotype with significant cardiac dysfunction and robust myocardial macrophage infiltration [72]. Innate and adaptive immune cells in the heart are complexly activated in TS-like cardiomyopathy, with macrophages being the predominant immune cells, as reported by results from single-cell RNA-Seq analyses of myocardial immune cells. They also demonstrated that mice exposed to ISO recovered from cardiac dysfunction after receiving either global macrophage depletion (through clodronate liposome injection) or blocking of macrophage infiltration (by a CCR2 antagonist or in CCR2-KO mice). ISO-induced cardiac dysfunction was also reduced when HIF1 deficiency or exposure to the immunomodulatory drug bortezomib dampened myeloid cell activation. Although the ISO-induced mouse cardiomyopathy model used to display TS seems an oversimplification of the exact clinical situation, these findings do represent a significant association that warrants further investigation into the possible role of macrophages.

### 3.6. Other Molecular Mechanisms

Some other molecular mechanisms are also implicated in BHS:

#### 3.6.1. Microvascular Reactivity and Coronary Spasm

Nuclear myocardial perfusion investigations have shown that apical perfusion is impaired at one and six months but eventually recovers [73]. Endothelin, catecholamines, and the related reactive oxygen species may be responsible for such microvascular dysfunction and aberrant perfusion. Another possibility is that myocardial inflammation causes direct myocyte damage, including vascular endothelial injury, which results in the shedding of the endothelium glycocalyx and subsequent myocardial edema [74]. However, there are concerns about cause and effect for both myocardial edema and microvascular dysfunction, which may be a result rather than a cause of the acute event. Wittstein proposed a link between sympathetic overactivity and microvascular dysfunction, as well as its impact on clinical presentation in patients with Takotsubo Syndrome. Individuals who are at high risk for microvascular ischemia and subsequent myocardial infarction have increased sympathetic tone and vasomotor dysfunction (postmenopausal status, depression, and therapy with serotonin reuptake inhibitors). Low-risk individuals with normal sympathetic and vasomotor tone will almost certainly require a significantly higher catecholamine surge to cause acute TS. This could explain why some patients appear after seemingly insignificant stimuli [75].

#### 3.6.2. Perfusion of the Apex 

In TS, the apex of the heart receives less perfusion than the base, causing one of the core problems in TS. This is because there is a considerable overexpression of structural genes at the apex of TS hearts compared to their bases or apexes in normal hearts. Moreover, there is also a large downregulation of metabolism-related genes [76].

#### 3.6.3. Inflammatory Mechanisms

During the acute phase, patients with TS have higher retention of ultra-small superparamagnetic iron oxide particles in both ballooning and non-ballooning left ventricular segments (TERRIFIC, NCT02897739). This may be due to the fact that ultra-small supramagnetic iron oxide particles are primarily phagocytosed by activated tissue-resident macrophages, and macrophages appear to be the primary cellular protagonists of myocardial cellular inflammation in acute Takotsubo Syndrome, whereas acute myocarditis is predominantly lymphocyte-mediated. Furthermore, it has been observed that serum interleukin-6 and chemokine (C-X-C motif) ligand 1 concentrations, as well as classic CD14+ CD16 monocytes, are elevated in patients with Takotsubo Syndrome, but intermediate CD14+ CD16+ and non-classic monocytes are decreased. At 5 months follow-up, the prevalence of M1 macrophages and the persistence of the intermediate (CD14+ CD16+) monocyte subgroup is highly suggestive of a less reparative and more pro-inflammatory state compared to patients with acute myocardial infarction at similar stages. It is unknown, however, whether this inflammatory activity causes or contributes to TS. Regardless, these findings shed light on the low-grade chronic inflammatory substrate that contributes to the progression of acute takotsubo into long-term heart failure [77,78].

## 4. Broken Heart, Why Not Happy Heart Syndrome?

The Broken Heart Syndrome is mostly associated with negative emotions and their impacts and thus is proclaimed to be the ‘Broken Heart’ syndrome. The significance of ‘positive emotions’ in TS is poorly defined [79,80]. Positive emotions and excessive happiness are just as endocrinologically powerful as negative emotions. These can modify the autonomic nervous system response leading to an altered heart rate, peripheral vascular resistance, and blood pressure. However, there are contradictory findings about the influence of good emotions on cardiovascular disease. Positive emotions have been linked to a lower risk of cardiovascular disease in the long run [81], although some data do suggest the contrary [82]. Positive emotions can trigger both the sympathetic and parasympathetic nervous system activity. Interestingly, in a predisposed individual, the chances of having a cardiovascular event on one’s birthday are 27% greater than on some other day which can directly be attributed to the associated positive stress [82]. Various cases have been reported of TS after some joyful or socially acceptable fun moment, highlighting that cardiomyopathy is stress-induced, which may either be positive or negative, and thus associating it with only negative emotions is nothing but an oversimplification. A systematic analysis by Ghadri et al. compared the ‘Broken Heart Syndrome‘ and ‘Happy Heart Syndrome and concluded that the baseline characteristics and clinical findings such as chest pain and dyspnea were comparable between the two syndromes, regardless of the nature of the triggering event. Moreover, the latter i.e., ‘Happy Heart’ group had greater prevalence of the midventricular type of TS. “Happy Heart Syndrome” makes up only 1.1% of all TS cases, implying that the ‘happy events’ may require more intense stimuli than negative emotions to elicit a significant emotional reaction and thus cause TS. When pleasant experiences are processed, the threshold for influencing the cardiovascular system may be higher [83].

## 5. Clinical Presentation

Takotsubo or stress-induced cardiomyopathy clinically manifests as an acute coronary syndrome. However, the onset of this condition is triggered by some intense emotional or physical distress or both. The most common physical exam findings for TS are acute substernal chest pain (75.9%), dyspnea (46.9%), and syncope (7.7%) according to the International Takotsubo Registry study [9]. Shortness of breath or dyspnea is thought to occur due to pulmonary edema. The syncope or dizziness can be due to hypotension or hypoperfusion [84]. Other symptoms include tachycardia, narrow pulse pressure, and systolic blood pressure less than 90 typically seen in hospitalized patients [85].

Patients can also develop complications such as a cardiogenic shock, which presents as hypotension, respiratory distress, diaphoresis, oliguria, and cold extremities seen in 10% of patients with TS [9]. Pulmonary hypertension can also occur which presents with jugular vein distention, RV heave, loud P2, cyanosis, dyspnea, syncope, lethargy, and fatigue. Other complications include severe mitral regurgitation which produces a holosystolic murmur and left ventricular outflow tract obstruction (LVOTO) which produces a late systolic murmur, S4, dyspnea, or syncope. LVOTO is thought to be induced by left ventricular basal hyperkinesis. Life-threatening arrhythmias such as AV block, asystole, fibrillation, tachyarrhythmias, brady-arrhythmias, and ventricular arrhythmias significantly worsen the prognosis. In worse cases, it can lead to stroke, sudden cardiac arrest, or heart failure. 

Takotsubo Syndrome has two distinct clinical forms: primary and secondary. Medical professionals encounter cases of Takotsubo Syndrome in a wide range of clinical settings. Primary Takotsubo Syndrome is the most common type, but secondary cases do occur. (1) *Primary Takotsubo Syndrome:* Patients with primary Takotsubo Syndrome typically visit an emergency room, a hospital with specialized cardiac care, or their primary care physician because they are experiencing acute cardiac symptoms. Some of these patients may or may not have easily recognizable sources of stress (often emotional). However, while comorbid conditions may increase the risk of experiencing a catabolization of catecholamines, they are not the primary cause. These patients have what is known as primary Takotsubo Syndrome, and the clinical management of their condition is contingent on the manifestations of the condition. (2) *Secondary Takotsubo Syndrome:* Most cases happen in people who are already hospitalized for treatment of something else, be it physical (such as an injury or illness), mental (such as depression), or procedural (such as labor or delivery). An acute Takotsubo Syndrome develops as a result of the primary condition or its treatment when the sympathetic nervous system is suddenly activated or when catecholamine levels are elevated. In these situations, we propose that a secondary Takotsubo Syndrome diagnosis be made. They need to have both the condition that brought on TS and its cardiac complications addressed in their care [8].

## 6. Principles of Management 

### 6.1. Diagnosis of Broken Heart Syndrome

**Figure 3:**
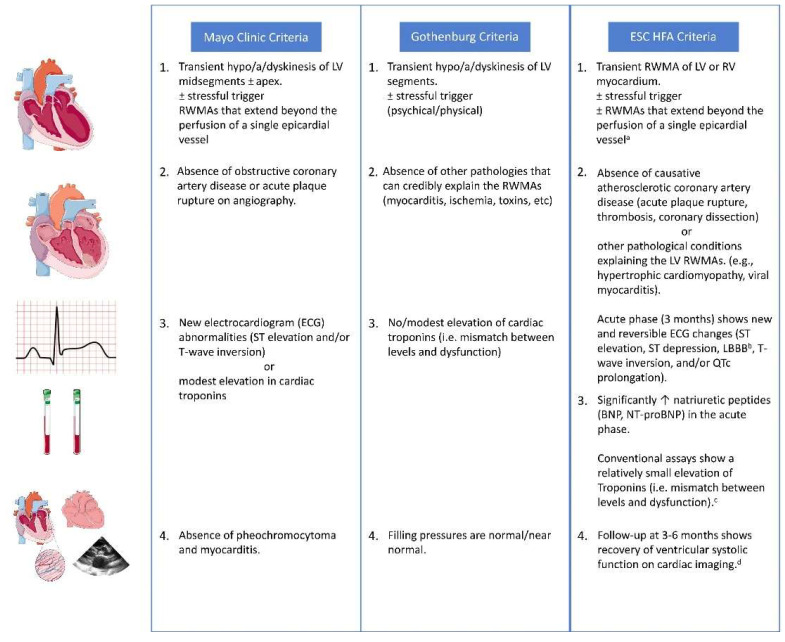
A comparison of various diagnostic Criteria. Mayo Clinic Criteria [93,94], Gothenburg Criteria [95], and ESC Heart Failure Association Criteria [8]. ^a^ Acute, reversible dysfunction of a single coronary territory. ^b^ Left bundle branch block may be permanent after Takotsubo Syndrome, but other cardiomyopathies should be excluded. ^c^ Troponin-negative cases are atypical. ^d^ Small apical infarcts and bystander subendocardial infarcts independently leading to regional wall motion abnormality is doubtful. ESC HFA: European Society of Cardiology Heart Failure Association. Figure attribution: Parts of the figure were drawn using pictures from Servier Medical Art (smart.servier.com), provided by Servier, licensed under a Creative Commons Attribution 3.0 unported license. (https://creativecommons.org/licenses/by/3.0/).

Broken Heart Syndrome has a very similar symptomatic presentation to ACS, and hence may be misdiagnosed. Therefore, in patients presenting with ACS-like clinical features, Broken Heart Syndrome should be considered as a possible differential diagnosis. Treatment as per ACS, with anticoagulation and fibrinolysis, poses a risk of serious bleeding complications, and even death in such patients. Similarly, misdiagnosis of BHS as cardiogenic shock due to LVOTO caused by extensive myocardial infarction and subsequent treatment with catecholamine inotropics can have disastrous results. In addition, patients lack any coronary lesion to explain the left ventricular wall motion abnormality (LVWMA) typically seen in TS. Therefore, prompt coronary angiography, including left ventriculography, is essential for detecting and differentiating TS from ACS. Moreover, it is imperative to mention here that ACS can also set off TS, so the two conditions are linked [86]. Alternative mechanisms of myocardial infarction with TS, such as coronary ulceration with peripheral embolization, transient coronary vasospasm, or spontaneous coronary artery dissection, should be considered in women presenting with ACS who do not have angiographically demonstrable obstructive coronary artery disease [87,88,89]. Non-invasive cardiac computed tomography angiography [87] may be an option for patients with severe comorbidities when invasive coronary angiography carries risks of complications. It is important to perform cardiac imaging studies, especially echocardiography, as soon as possible after patient presentation and to repeat these studies, as the LVWMA may resolve in hours to weeks [86] (Figure 3). Additionally, echocardiography is helpful in determining the location of LVWMA, potential right ventricle involvement, and TS complications such as LV thrombus and LVOTO are present. The morphology of TS, the involvement of the right ventricle, the presence or absence of myocardial edema, and the presence or absence of TS complications such as left ventricular thrombus are all invaluable pieces of information that can be gleaned from CMR imaging. Myocardial infarction and myocarditis are two examples of diseases that can cause irreversible damage to the heart, but CMR imaging can tell them apart from TS [90,91].

Thus, it becomes very important to have clinical clarity in the diagnosis of Broken Heart Syndrome and to have a high index of suspicion. This is largely helped by diagnostic criteria such as the Mayo Clinic Criteria [92,93], Gothenburg Criteria [94], and ESC Heart Failure Association Criteria [8] (Figure 3).

### 6.2. Differential Diagnosis

It is important for timely diagnosis that other differentials are well-demarcated and that differences are cleanly understood. Table 1 provides a comparison between most common differential diagnosis for TS.

### 6.3. Risk Stratification

TS can lead to life-threatening complications, the most common being Acute Heart Failure (12–45%) and thus requires close observation and risk assessment [91]. Multiple attempts have been made to stratify patients with TS according to a risk score to further guide management. The Mayo Clinic Risk Score, was developed and validated in 2011 by Madhavan et al., assigning 1 point to three major risk factors (Age > 70 years, presence of physical stressor and EF <40%) for the development of Acute Heart Failure in patients with TS. The risk of Acute HF is around 28%, 58%, and 85% with a score of 1, 2, and 3, respectively. The goal was to triage high-risk patients to an intensive care unit for initial management and identify those patients where inotrope and beta-blockers use may be harmful [92].

**Figure 4:**
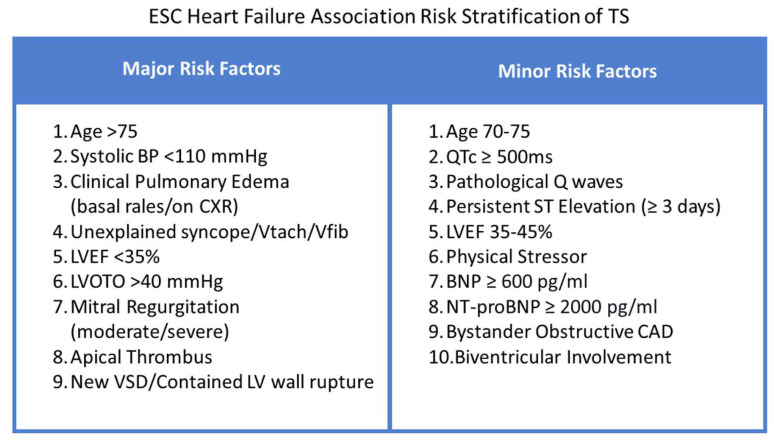
European Society of Cardiology (ESC) Heart Failure Association Risk Stratification of TS [8].

Lyon et al., 2016, suggested a more comprehensive risk stratification score based on major and minor risk factors to help guide management (Figure 4). The management algorithm differed based on whether the patient was classified as low or high risk [8].

### 6.4. Biomarkers

Currently, natriuretic peptides and troponins are recommended biomarkers that help in reliably differentiating TS from ACS [9]. Both natriuretic peptide levels are much higher than that seen in myocardial infarction [93]. Research shows that B-type Natriuretic Peptide (BNP) has been consistently elevated in patients with TS, and the elevation correlates with the degree of wall motion abnormality. NT-proBNP, an inactive N-terminal molecule of BNP, is also typically elevated [94,95]. Peak levels are obtained 24–48 h after presentation and resolve within 3 months [94]. In a study by Wittstein et al., the levels fell rapidly and by day 7, 8, or 9, were lower than patients with MI, correlating with a rapid improvement in left ventricular function [96]. Atypical types of TS present with a lower elevation compared to the typical apical ballooning type of wall motion abnormality, which is associated with more severe left ventricular dysfunction [1]. Apart from natriuretic peptides, cardiac troponins are measured as well. Median cardiac troponin levels on admission are 7.7 times the upper limit of normal in patients with TS and comparable to ACS, but peak levels are lower in TS compared to STEMI probably due to the limited role of myocardial necrosis in its pathogenesis [9,97]. Since BNP and Troponins tend towards opposite directions in TS compared to STEMI, BNP/TnI (Troponin I) ratio has emerged as a more reliable diagnostic tool than either of them alone [93].

Catecholamines, their precursors and degradation products are also significantly raised in some patients, although they are not routinely used in clinical practice [96]. Creatine kinase levels are typically not significantly elevated [9]. Recently, microRNAs have emerged as an additional diagnostic tool for supporting the diagnosis of TS [30]. 

#### 6.4.1. Other Biomarkers

Many studies have demonstrated an increase in pro-inflammatory biomarkers with the onset of TS. Scally et al. demonstrated the presence of a monocytic infiltrate in both the ballooning and non-ballooning segments, an increase in pro-inflammatory cytokine levels (especially interleukin 6 and CXCL1), and a change in the distribution of monocyte subsets. A few of these changes have seen to persist for at least 5 months, indicating chronic inflammatory changes [78].

However, IL-6 levels have been shown to be significantly higher in patients with ACS.13 In fact, Santoro et al. found that TS was characterized by an increase in anti-inflammatory cytokines (IL-2, IL-4, and IL-10) in the acute phase [98]. This is probably due to macrophagic infiltrates, which are able to induce and inhibit inflammation. The significantly higher increase in IL-6 in ACS was attributed to the role of atherosclerosis in its pathogenesis. IL-6 is concentrated in the shoulder region of the plaque, which is prone to rupture and hence releases IL-6 in large amounts during ACS [99,100]. Although the differences in interleukin levels highlight the distinct pathogenesis of TS as compared to ACS, it is of questionable diagnostic utility as the differences were small.

Catecholamine-induced myocardial stunning has emerged as a major proposed pathogenesis of TS [27]. Reports of sympathomimetic drugs inducing TS without any emotional and physical triggers further support this hypothesis [101,102,103]. Wittstein et al. found that the levels of Norepinephrine, Epinephrine, and Dopamine were elevated to significantly higher levels in patients with TS compared to patients with Killip class III myocardial infarction. Plasma levels of dihydroxyphenylalanine, dihydroxyphenylglycol, and dihydroxyphenylacetic acid were twice the levels seen in the MI patient group, reflecting an increase in the synthesis of catecholamines, neuronal reuptake, and metabolism, respectively. The levels of catecholamine metabolites such as metanephrine and normetanephrine were also proportionately raised [96].

Other studies have shown that the increase in norepinephrine is not consistently seen in all patients with TS. Akashi et al. observed that around half of the patients studied did not show a significant elevation. The elevation could have also been a result of acute heart failure induced by TS, but the authors concluded that the very high catecholamine levels and patterns of regional sympathetic activation seen in some patients pointed towards a direct correlation [104]. Neuropeptide Y and Serotonin were also found to be significantly elevated in the days following admission in patients with TS compared to Killip class III MI [96].

#### 6.4.2. Novel Biomarkers 

MicroRNAs have emerged as important mediators in the pathogenesis of TS. In a study by Couch et al., miR-16 and miR-26a were found to enhance the negative inotropism of adrenaline on apical cardiac myocytes and positive inotropism on the basal segment. These microRNAs are also associated with anxiety and depression, potentially explaining the role of preexisting mental illness in increasing the vulnerability of the myocardium to an acute emotional trigger [67]. MiR-1 and miR-133a were found to be early markers of various cardiovascular diseases including STEMI and TS, indicating cardiomyocyte death [105]. However, a more recent study by Jaguszewski et al. found that the levels were much higher in STEMI compared to TS. Cardiac-specific miRs (miR-1 and miR-133a) were found to be significantly more elevated in STEMI and miRs associated with neuropsychiatric disorders (miR-16 and miR-26a) were significantly higher in TS. The combination of all four markers allowed for greater discriminating power, leading to a unique 4-panel miR signature that reliably differentiated between TS and STEMI (sensitivity 96.77%, specificity 70.37%). A fall in endothelin-1 (ET-1)-regulating miRNA-125a-5p levels was also observed, in tandem with a sharp rise in ET-1 plasma levels [30].

### 6.5. Electrocardiography

The ECG in a patient with TS initially shows ST-elevations in the precordial leads, the distribution of which is dependent on the regional pattern of ventricular ballooning. ST elevations were the most common finding in the InterTAK Registry study (44% of patients), followed by T wave inversions (41%), ST-segment depression (8%) and left bundle branch block (5%) [9]. Rarely, there may be no changes seen on the ECG. The findings depend on the time elapsed between the onset of symptoms and ECG measurement. The initial ST elevations are followed by T wave inversions and QT prolongation. QT prolongation resolves by day 14, but T wave inversions may persist up to 1 year after the event [106]. Four phases have been described by Mitsuma et al., Phase 1: ST elevations, Phase 2: T wave inversions (days 1 to 3), Phase 3: Transient resolution of T wave inversions (days 2 to 6) and Phase 4: Appearance of prominent, deep T wave inversions and QTc prolongation [107]. An ECG can support the diagnosis of TS but cannot confirm it; rather, a coronary angiogram is ultimately required for diagnosis [108].

#### 6.5.1. ST Segment

ST segment elevation (STe) over the precordial leads is a common finding on admission. They tend to be less prominent than those seen in STEMI. However, the V1 lead is less likely to be involved as it denotes the anterior and paraseptal right ventricular walls, which TS rarely extends to. The diffuse nature of ST elevations is probably due to the diffuse involvement of the ventricular wall, extending beyond the region supplied by a single vessel. The -avR lead points to the left ventricular apex (+30°, frontal plane), and frequently shows ST elevations, reliably differentiating it from ACS. In fact, it is a common fixture in the multiple algorithms developed by Frangieh et al. for differentiating ST elevation TS from STEMI. This study found that ST elevations in -avR along with ST elevations in anteroseptal leads (more than 2 out of V1, V2, V3) had a 100% specificity and positive predictive value for diagnosing ST elevation TS. On the other hand, ST depressions in V2, V3, and V4 had a 100% specificity for diagnosing STEMI [109]. However, a coronary angiogram is still required to reliably differentiate between the two [110]. Another algorithm was developed by Mugnai et al. using three criteria: ST depression in avR, no ST elevation in V1, and no abnormal Q waves, which had a specificity of 95% and a positive predictive value of 85.7% [111]. The STes in TS generally involve leads V2-V5 and the inferior leads II and avR whereas STEMI tends to involve leads V1-V4 and lateral leads I and avL [91]. Isolated STes in II, III avF is very rare in TS and could point to a different diagnosis [109]. Three different morphologies of the STes have been observed: concave, straight and convex. Piackova et al. suggest that the straight type STes might indicate a poorer prognosis [112]. ST depressions are less common in TS compared to ACS and can be used as a differentiating characteristic (around 10% in TS vs. 30% in ACS) [9]. Curiously, reciprocal ST depressions are also generally absent in TS [113].

#### 6.5.2. T Wave

T wave inversions, without ST elevations, may be present in patients who are evaluated later in the disease course. They closely accompany ST elevations, following a similar pattern of distribution but more widespread [114]. T wave inversions can persist up to a year after the initial event, acting as a marker of a prior episode of TS, similar to STEMI [106]. Myocardial edema appears to be the underlying cause of the inversion, along with lower QRS voltages (<5 mm in the limb leads and <10 mm in the precordial leads) (lQRS). A transient attenuation of the QRS complex has been observed (attQRS) in various case reports, probably due to the decreased electrical conduction through the edematous myocardium [115,116].

#### 6.5.3. QT Segment 

QT prolongation develops around 24–48 h after the trigger (when present) or initial presentation, which is not commonly seen in STEMI and may be a helpful distinguishing feature. The prolongation is often significant (>500 ms) and predisposes the patient to life-threatening ventricular tachyarrhythmias and hence is an important risk stratification tool [117,118]. Other less common findings include LBBB, ST depressions, ST elevation in V1, Pathologic Q waves, J waves with QRS aberrations, etc. [91,119,120,121]. Pathologic Q waves are rarely observed in TS and resolve within 1 month. Anterior Q waves without ST elevations or T wave inversions may be present in a minority of patients, referred to as ‘anterior infarction, age indeterminate’ [91].

### 6.6. Coronary Angiography and Ventriculography 

A coronary angiogram is a crucial diagnostic step to dependably differentiate TS from ACS. Most patients present with chest pain and ST elevations on ECG, along with raised cardiac enzymes, and almost invariably require urgent coronary angiography. Usually, no obstructed coronary arteries are found, however, they may coexist in around 15% of patients [9,122]. The absence of obstructive CAD was included in the Mayo Clinic Diagnostic Criteria but has since been modified [123]. Non-obstructive CAD was previously hypothesized to be a cause of TS, with transient coronary thromboembolism acting as the initial trigger. The current understanding suggests that it is simply a coexistence, but it is associated with a higher risk of heart failure [8,124].

In the event of TS coexisting with obstructive CAD, biplane ventriculography should be utilized. The ventricles should be visualized at the same angle with careful observation for a wall motion abnormality not consistent with the distribution of the affected arteries [125,126]. Prior to conducting ventriculography, plaque rupture, thrombosis, and coronary dissection must be excluded. Characteristic wall motion abnormalities may be missed if it is delayed beyond a few hours [8]. “Apical nipple sign” refers to an unaffected region of the ventricular apex that contracts normally, observed in around a third of patients with TS [127]. Ventriculography is often diagnostic for TS. LVOTO is a common complication of TS (around 20%), therefore pressure gradients in the outflow tract should be measured [128]. Left ventricular end-diastolic pressure (LVEDP) is also an important measurement, and high values may indicate acute heart failure, which is the most common complication of TS. A total of 93% of patients in the InterTAK Registry Study had elevated (>11 mmHg) LVEDP on angiography [9].

### 6.7. Echocardiography 

**Figure 5:**
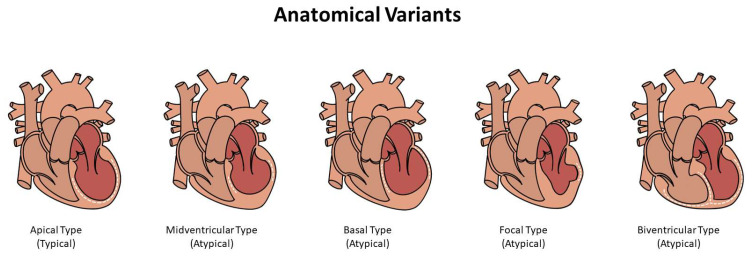
Anatomical Variants of TS (Typical and Atypical forms).

The most important non-invasive imaging modality is the Transthoracic Echocardiogram with color and tissue doppler. Left ventricular ejection fraction (mean value, 40.7 ± 11.2%) was reduced in 86.5% of TS in the acute phase, compared to 54.2% of patients (mean value, 51.5 ± 12.3%) with acute coronary syndrome [9]. Echocardiography can detect a variety of variants (Figure 5) such as mid-apical myocardial segment ballooning, hypo-, a-, or dyskinesia. Other findings may also be observed, including involvement of the inferior or midventricular anterolateral wall, as well as the anterior or entire interventricular septum. On 2D speckle-tracking imaging, LV twisting is either reduced or flipped to clockwise apical rotation in the acute phase, and the rate of untwisting is slowed down, making it a more sensitive index of regional diastolic dysfunction. Hypokinesia, akinesia, or dyskinesia of the midventricular segments characterize transient tachycardia with tachycardia (TS). Forms involving only the basal segments, or “basal forms,” This phenotype is uncommon and is seen most often in patients with phaeochromocytoma, epinephrine-induced TS, or subarachnoid hemorrhage [3,129,130,131,132].

### 6.8. Cardiovascular Magnetic Resonance (CMR) Imaging

CMR offers a high diagnostic sensitivity for detecting myocardial inflammation in patients with TS. The anterolateral segment is most commonly affected by focal TS. CMR is necessary to differentiate this rare form of TS from ACS or myocarditis. In its most basic form, right ventricular involvement manifests as dilatation of the RV with hypo- to akinesia of the free wall and apex. As a result of the abnormalities in LV wall motion caused by TS go beyond the territory supplied by any one coronary artery, the systolic dysfunction seen on speckle-tracking echocardiography appears circular. When more than four segments of the WMSI are dysfunctional, TS can be diagnosed with a sensitivity of 83% and specificity of 100% [3,91,129,130,131,132,133,134].

## 7. Treatment Modalities

Supportive care and the management of complications and comorbidities make up the bulk of treatment. The next focus is on preventing complications and supporting recovery. Since an initial clinical differentiation between ACS and TS is difficult to make, suspected patients should be started on treatment according to the ACS guidelines, particularly morphine, oxygen, heparin, and aspirin [91]. Given the high risk of arrhythmias in these patients, continuous ECG monitoring is recommended [84]. A trans-thoracic echocardiography can then be recommended to rule out a Left Ventricular Outflow Tract Obstruction (LVOTO). An older age, septal bulge, and hemodynamic instability may point out to LVOTO [128]. If detected, the use of inotropes needs to be avoided in treatment. In cases of moderate to severe LVOTO, a beta-blocker is recommended to alleviate the obstruction.

Further treatment is better individualized as per the hemodynamic stability of the patient. For hemodynamically stable patients, manage systolic heart failure. The main drugs indicated are ACE inhibitors (such as lisinopril) and low-dose beta-blockers (such as metoprolol tartrate). For hemodynamically unstable patients the treatment is dictated by the status of LVOT obstruction. In patients with no LVOT obstruction, there are a few options for inotropic support, including dobutamine, dopamine, and even levosimendan, but it is important to keep in mind that all three can cause tachycardia and worsen Takotsubo cardiomyopathy. Patients on inotropic therapy need close observation for the emergence of LVOT. If inotropes are ineffective, vasopressors can be used as a backup. It is important to think about advanced treatments when traditional methods have failed; high-pressure balloon catheter inserted into the aorta (IABP) and devices for ECMO assistance of the left ventricle form the terminal resort. In some cases (up to 25%), the LVOT becomes obstructed. In addition to being difficult to treat, LVOT obstruction reduces the LV’s ability to pump blood. If the LVOT is blocked, inotropic support should be avoided because it can lead to cardiogenic shock. Increasing systolic function of the left ventricle (LV) may be attainable with intravenous fluids. If tolerated, a short-acting, low-dose beta blocker may help alleviate LVOT obstruction, though its use should be cautious in patients with hypotension. A mechanical aid for the heart’s failing left ventricle or advanced treatments maybe considered as a last resort. Patients may also require ECMO support [84,135,136].

### 7.1. Evolving Concepts for Heart Failure

Stress cardiomyopathy patients undergoing treatment for HF focus on decongestive therapy and hemodynamic support for states of low cardiac output. Patients with pulmonary congestion who do not have hypotension or low cardiac output are treated with venodilators (such as nitroglycerin, nitroprusside, or nesiritide) and diuretic agents to decrease venous return. Patients with systemic arterial hypertension may benefit from the use of arterial vasodilators; however, they must be monitored closely for worsening of left ventricular outflow tract obstruction (LVOTO). When treating hypertension, low-dose beta-adrenergic receptor blockers may be helpful in cases of LVOTO due to their ability to decrease basal hypercontractility and, consequently, relieve obstruction in the heart’s aortic valve. As a bridge to mechanical support with a left ventricular assist device or to recovery, vasopressor drugs (i.e., phenylephrine, norepinephrine, or vasopressin) should be considered if inotropic agents are insufficient. Patients in shock who develop LVOTO with or without MR present a significant therapeutic challenge. Since they have the potential to increase basal hypercontractility, inotropic agents should be avoided in cases where obstruction is already a problem. If the patient is already taking inotropic medications, it may be beneficial to reduce the dosage or suspend the medication and administer intravenous fluids in order to facilitate the clearance of the obstruction. A low dose of a short-acting beta-adrenergic receptor blocker (e.g., esmolol, metoprolol) can be tried to reduce the LVOTO if it is severe and the patient is not bradycardic, with the hope of improving cardiac output. Peripherally acting vasopressor drugs (i.e., phenylephrine or vasopressin) may be indicated in the treatment of shock with LVOTO because they raise blood pressure without raising LV obstruction; however, if they fail to improve LVOTO, these agents may worsen cardiac output. A mechanical cardiac assist device should be considered if medical treatment fails to improve cardiac function. Extracorporeal membrane oxygenation is commonly used to aid patients experiencing severe shock [84].

### 7.2. Current Evidence for Pharmacotherapy 

ACE inhibitors and ARBs are said to promote the recovery of the left ventricle. Beta-blockers are proposed to act by attenuating the apical ballooning [137]. Singh et al. in a meta-analysis found ACEi to be more beneficial rather than beta-blockers to prevent recurrence of TS [10]. Another potential negative for beta-blockers is the possibility of QT prolongation that might worsen the condition. Once LVOTO has been ruled out, further treatment depends on the type of pathology. Diuretics and nitroglycerin can be used in cases of heart failure and pulmonary edema. Inotropes such as Dopamine and Dobutamine are used in systolic failure when LVOTO is ruled out [91]. Levosimendan is a calcium sensitizer that can be used as an alternative inotropic agent. A case series by Santoro et al. on 13 TS subjects found Levosimendan to be safe and feasible with just 15% of cases reporting adverse effects [138]. Low-dose vasopressors can only be used temporarily as an adjunct to help in recovery [84]. In cases of LVOTO that present with shock, the aim is to increase BP without impacting the LVOTO. Thus, peripherally acting vasopressors are used (Phenylephrine, Vasopressin).

Since aldosterone further enhances the cardiovascular effects of systemic catecholamines, an Aldosterone receptor blocker (Spironolactone) may be used. Angiotensin receptor-neprilysin inhibitors may also be used in cases of heart failure [139]. Beta-blockers may be considered for arrhythmia prevention. Heparin, Vitamin K antagonists or Novel Oral Anticoagulants are recommended for the prevention of thromboembolism. Anticoagulation is considered in cases of reduced ejection fraction (<= 30%) or a Large Left Ventricular Dysfunction involving the cardiac apex. Chronic treatment of TS involving the use of beta-blockers, ACEi, ARBs, and Calcium Channel blockers have shown variable results in studies [84]. A meta-analysis involving 511 patients with 23 recurrence instances showed no significant benefit of beta-blockers, ACEi, ARBs, Statins, and Aspirin in preventing TS recurrence. An ongoing prospective, randomized-controlled trial experimenting with the use of N-acetylcysteine (NAC), followed by/or oral ramipril for 12 weeks in the treatment by assessing a decrease in myocardial edema and improvement in the LV systolic function [140]. Attention should also be given to the patient’s mental health, given the strong association of anxiety and mood spectrum disorders in these patients [141].

## 8. Complications

Broken Heart Syndrome does not end with an acute episode and the left ventricular function may return to normal within a few weeks, but several complications may occur before systolic function recovers pushing the in-hospital mortality rate is as high as 5% [84]. These complications include (but are not limited to): 

### 8.1. Cardiogenic Shock and Acute Heart Failure 

Twelve percent to forty-five percent of patients develop systolic HF during the acute phase [129,142]. Old age, low left ventricular ejection fraction (LVEF), high troponin levels, a midventricular pattern, RV involvement, and a physical (as opposed to emotional) stressor are all independent risk factors contributing to poor prognosis. Acute HF manifests with symptoms such as dyspnea, lightheadedness, fainting spells, tachycardia, and lactic acidosis. There are a number of complications that can lead to acute HF, including LVOTO and MR. The echocardiogram reveals a severely decreased stroke volume due to a decreased LVEF in a non-dilated LV, even in the absence of complications [143].

### 8.2. Arrhythmias

Nearly a quarter of patients experience arrhythmias. Five percent to fifteen percent of patients experience atrial fibrillation, and this condition is linked to decreased LVEF and an increased risk of cardiogenic shock [144]. In the acute phase, 4% to 9% of patients experience ventricular arrhythmias [145,146]. When the QT interval is prolonged by more than 500 milliseconds, stress cardiomyopathy can lead to a potentially fatal torsade de pointes [147]. When compared to tachycardia, other arrhythmias occur much less frequently. AF on admission is significantly associated with increased in-hospital and long-term mortality rates. [148]. An increased risk of arrhythmias appears to exist until ECG abnormalities such as T-wave inversion, QT prolongation, and/or LVEF depression improve. Atrial fibrillation (AF) has been found to be a significant contributor to mortality in patients with Takotsubo cardiomyopathy. According to a global, multi-center study of 387 patients, patients with Takotsubo cardiomyopathy and AF had a higher long-term mortality rate than those without AF. In-hospital mortality was higher in patients with Takotsubo cardiomyopathy and AF (19%) compared to those with Takotsubo cardiomyopathy but no AF (10%) [149]. Patients with Takotsubo cardiomyopathy and AF have an increased risk of mortality, and this increased risk may have multiple causes. Even though thromboembolic events are a known risk of AF, they account for only about 1% of deaths in patients with Takotsubo cardiomyopathy and AF [146].

### 8.3. Obstruction of the Left Ventricular Outflow Tract

In the classic apical ballooning forms, LVOTO and mitral valve systolic anterior motion due to the Venturi effect cause MR in 14% to 25% of patients [150] due to hypercontractility of the basal LV segments. Due to the additional obstruction of LV systole caused by the presence of LVOTO and MR, these cases are the most severe and the most challenging to treat. To avoid making the obstruction and cardiogenic shock even worse, it is crucial to rule out LVOTO. Ejection systolic murmurs with loud S2 are suggestive of obstruction, which can be seen on Doppler echocardiography or pressure waveform assessment during pull-back after ventriculography. Hemodynamic significance is defined as an instantaneous gradient greater than 25 mm Hg, and a high risk is indicated by a gradient of less than 40 mm Hg.

### 8.4. Thrombo-Embolism 

Stroke and embolization can result from LV thrombus formation (which occurs in 2% to 9% of cases) [144,151]; this is especially true for apical forms of LV thrombus formation. When LV function is already low, the risk of thrombi is highest, somewhere between days 2 and 5 after the onset of symptoms [152]. Patients diagnosed with stress cardiomyopathy should undergo follow-up echocardiography because thrombus may resolve after 2 weeks of anticoagulation; however, a late occurrence has also been described. Up to 17% of patients with LV thrombi will experience a cerebrovascular embolic event [152]. Stress cardiomyopathy patients with apical ballooning or other large areas of wall motion abnormality should be considered for systemic anticoagulation until LVEF recovers [84,151].

### 8.5. Intramyocardial Hemorrhage and Rupture 

Patients with stress cardiomyopathy have been reported to experience intramyocardial hemorrhage and ventricular wall rupture, both of which are indicative of severe ischemia-reperfusion injury [153]. The risk of bleeding and rupture increases with age, hypertension, persistent ST-segment elevation, and infrequent beta-blocker use [154].

## 9. Prognosis

For a long time, TS was thought to be a non-life-threatening condition; however, recent research has shown that TS is a potentially fatal illness with high morbidity and mortality during the acute phase and outcomes that are comparable to those of ACS [155]. Mortality rates are higher for male patients than for female patients, especially for those with underlying critical illness [156]. Depending on the specific set of circumstances that precipitate it, TS can be anything from a mild inconvenience to a potentially lethal disease. Therefore, there are many more facets to TS than are initially apparent. A positive prognosis is shown for “the classic patient with TS,” an elderly woman with an emotional trigger event and apical ballooning, while patients with TS secondary to neurologic disorders and TS secondary to physical activities, medical conditions, or procedures show an unfavorable outcome [155]. A meta-analysis performed by Singh et al. [157] found that patients with TS who also had underlying noncardiac conditions had a higher rate of in-hospital mortality. These patients with varying physical triggers had varying short- and long-term outcomes.

## 10. Future Perspectives

The exact etio-pathogenesis for TS is still obscure, but over the years of extensive research, we have made significant leaps and have framed the treatment for the disease. Many of the molecular mechanisms underlying TS have been elucidated through the integration of genomics, proteomics, transcriptomics, metabolomics and mircobiomics which may aid in the advancement of precision medicine. The implementation of various ‘omics’ can help translate individualized and precise helping better prognosis and lesser complications. Development and provision of suitable treatment options for each patient require knowledge of the molecular mechanisms by which each mutation contributes to disease pathogenesis. New technological developments, such as an innovative approach to systematically assessing the functional significance of multiple variants in vivo, would be required to achieve this goal. Furthermore, technologies such as artificial intelligence can help in risk stratification, while computational cardiology models can be employed to prevent the recurrence of complications [158,159].

## 11. Conclusions

Broken Heart Syndrome or Takotsubo Syndrome (TS) is a condition that manifests clinically as an acute coronary syndrome (ACS). It is characterized by hypokinesia of the basal segment and various other wall motion abnormalities. It is believed to be a consequence of several psychiatric and neurological conditions. The pathophysiology of this condition is elusive, the specificities of which are still being investigated, which makes the diagnosis and treatment more strenuous. The recent growth in molecular evidence has laid foundations for future research which will help establish new drug targets helping us a step closer to precision medicine. 

## Figures and Tables

**Table 1:** Differential Diagnosis of Broken Heart Syndrome and their clinical presentation.

Disease	Clinical Presentation	Electrocardiography	Echocardiography	Coronary Angiography	Cardiac Magnetic Resonance	Biomarkers
Stress Cardiomyopathy	Chest pain (mostly substernal), dyspnea, syncope, tachycardia, arrhythmias (tachyarrhythmias or bradyarrhythmia), mitral regurgitation or sudden cardiac arrest.	ST-segment elevation, T-wave inversion, QTc prolongation	Hypo/ akinesia in apical, basal, midventricular, or focal regions	Evidence of obstructive CAD and acute plaque rupture are absent on angiography	Edema: Dysfunctional ventricular myocardium shows transmural edema. Cine-CMR: shows RWMAs depending on the anatomical variant DGE absent (cut-off >5 SD)	NT-proBNP and BNP are severely elevated. Troponins and CK-MB are mildly elevated.
Myocardial Infarction	Chest pain (usually at the center of the chest “*Levine sign*” and radiating to an upper extremity, particularly arms, shoulder, and lower jaw), diaphoresis, nausea/vomiting, dyspnea, malaise, arrhythmias, sudden cardiac death.	ST-segment elevation, ST-segment depression and/or T-wave inversion	RWMAs correspond to the vascular distribution of epicardial coronary arteries involved.	CAD with acute plaque rupture, thrombosis, or coronary dissection	Edema: Can be subendocardial or transmural at the locations of RWMAs. Cine-CMR: RWMAs correspond to the vascular distribution of epicardial coronary arteries involved. DGE: Affected regions show bright DGE in subendocardial or transmural patterns and correspond to vascular distribution of involved coronary arteries.	Troponin and CKMB levels markedly elevated. BNP and NT-proBNP mildly elevated
Myocarditis	Chest pain, dyspnea, excessive fatigue or exercise intolerance, unexplained sinus tachycardia and respiratory distress/tachypnea. May lead to acute heart failure and sudden cardiac death. Preceding upper respiratory infection or enteritis is often present.	ECG can be normal or have non-specific findings such as ST–T-wave changes (myopericarditis typically shows diffuse ST elevations)	Global systolic dysfunction (can sometimes be regional or segmental), LV dilation, changes in LV geometry, and wall motion abnormalities. The pericardium may also be involved.	Evidence of obstructive CAD and acute plaque rupture are absent on angiography	Edema: Distribution is subepicardial, lateral, or basal Cine-CMR: Generally global dysfunction except when focal edema is severe. DGE: Subepicardial, midventricular or focal “patchy” low intensity or bright DGE. Distribution does not correspond to coronary vascular patterns.	Troponin may or may not be elevated. CK-MB mildly elevated. BNP and NT-proBNP are mildly elevated. Acute phase reactants (like ESR, CRP) elevated.

DGE: Delayed Gadolinium Enhancement.

## Data Availability

Not applicable.
